# Economic Impact of a New Rapid PCR Assay for Detecting Influenza Virus in an Emergency Department and Hospitalized Patients

**DOI:** 10.1371/journal.pone.0146620

**Published:** 2016-01-20

**Authors:** Marcelo Soto, Laura Sampietro-Colom, Anna Vilella, Efraín Pantoja, María Asenjo, Ruth Arjona, Juan Carlos Hurtado, Antoni Trilla, Míriam José Alvarez-Martínez, Aurea Mira, Jordi Vila, María Angeles Marcos

**Affiliations:** 1 Fundació Clínic per a la Recerca Biomèdica, Barcelona, Spain; 2 Health Technology Assessment Unit, Hospital Clínic, University of Barcelona, Barcelona, Spain; 3 Public Health Department, Hospital Clínic, University of Barcelona, Barcelona, Spain; 4 ISGlobal Barcelona Institute for Global Health, Barcelona, Spain; 5 Emergency Department, Hospital Clínic, University of Barcelona, Barcelona, Spain; 6 Department of Clinical Microbiology, Biomedical Diagnostic Centre (CDB), Hospital Clínic, University of Barcelona, Barcelona, Spain; 7 Biomedical Diagnostic Centre (CDB), Hospital Clínic, University of Barcelona, Barcelona, Spain; University of Calgary & ProvLab Alberta, CANADA

## Abstract

Seasonal influenza causes significant morbidity and mortality and has a substantial economic impact on the healthcare system. The main objective of this study was to compare the cost per patient for a rapid commercial PCR assay (Xpert^®^ Flu) with an in-house real-time PCR test for detecting influenza virus. Community patients with influenza like-illness attending the Emergency Department (ED) as well as hospitalized patients in the Hospital Clínic of Barcelona were included. Costs were evaluated from the perspective of the hospital considering the use of resources directly related to influenza testing and treatment. For the purpose of this study, 366 and 691 patients were tested in 2013 and 2014, respectively. The Xpert^®^ Flu test reduced the mean waiting time for patients in the ED by 9.1 hours and decreased the mean isolation time of hospitalized patients by 23.7 hours. This was associated with a 103€ (or about $113) reduction in the cost per patient tested in the ED and 64€ ($70) per hospitalized patient. Sensitivity analyses showed that Xpert^®^ Flu is likely to be cost-saving in hospitals with different contexts and prices.

## Introduction

Influenza is an important public health problem worldwide. Annual seasonal influenza epidemics are associated with a high rate of hospitalization and mortality, and considerable demand on healthcare resources [1–4]. Influenza infection usually results in mild and self-limiting respiratory symptoms but may also be responsible for severe disease, especially in chronic patients. Early diagnosis is essential since test results determine the management of patients and, ultimately, their health outcomes [5–7].

Several molecular tests have become the reference methods for the detection of influenza virus in clinical microbiology laboratories due to their high level of sensitivity and specificity [8,9]. However, such tests still require the use of specialized equipment, extensive hands-on time and have an average analytical turnaround time of 3 to 4 hours.

The Section of Virology of the Department of Clinical Microbiology of the Hospital Clínic (Barcelona, Spain) is a National Influenza Centre of the World Health Organization (WHO) for monitoring influenza virus infection. Prior to 2014, specimens were tested for influenza using an in-house real-time PCR assay that required 4 hours of technical expertise; as such, it was only carried out during working hours (i.e. from 9 am to 3 pm five days per week), thereby delaying the time of result delivery. This, in turn, affected the time to discharge of ED patients and unnecessarily increased the isolation time for hospitalized patients.

The Xpert^®^ Flu assay (Cepheid, Sunnyvale, CA) is an automated, multiplex real-time RT-PCR test for qualitative *in vitro* detection and differentiation of influenza A, influenza B, and influenza A subtype 2009 H1N1. It is well adapted to point-of-care laboratories because of its ease of use and less than 2 minutes of hands-on time. This test yields results in 70 minutes allowing sample processing upon arrival to the Microbiology Laboratory, providing rapid results 24 hours a day, 7 days a week. Previous validation studies of Xpert^®^ Flu have reported a sensitivity and specificity of greater than 90% [10].

The objective of the present study was to perform a cost analysis of the implementation of the Xpert^®^ Flu test compared to an in-house real-time PCR test to detect influenza virus in patients with influenza-like illness (ILI) admitted to the ED and in hospitalized patients.

## Methods

This is a cohort study including a control group consisting of patients tested for influenza between January and March 2013 and a treatment group prospectively recruited from January to March 2014. Adult patients (>18 years) attending the ED of the Hospital Clinic with ILI [11] and considered at high risk for influenza complications [12] were included in the study. Hospitalized adult patients with ILI were also included. Duplicate specimens from the same patient were excluded. In 2013, samples were exclusively tested by our in-house real-time PCR [13,14]. In 2014, all the samples were analyzed in parallel by the in-house real-time PCR and the Xpert^®^ Flu assay. Discrepant results were confirmed by DNA sequence analysis [15] (see Molecular Detection A in S1 File). Agreement between the two tests was assessed using the Kappa coefficient [16]. Management of patients from the treatment group was based on the results of Xpert^®^ Flu. Data were analyzed anonymously. This study was approved by the Ethics Committee of the Hospital Clinic of Barcelona.

**Fig 1:**
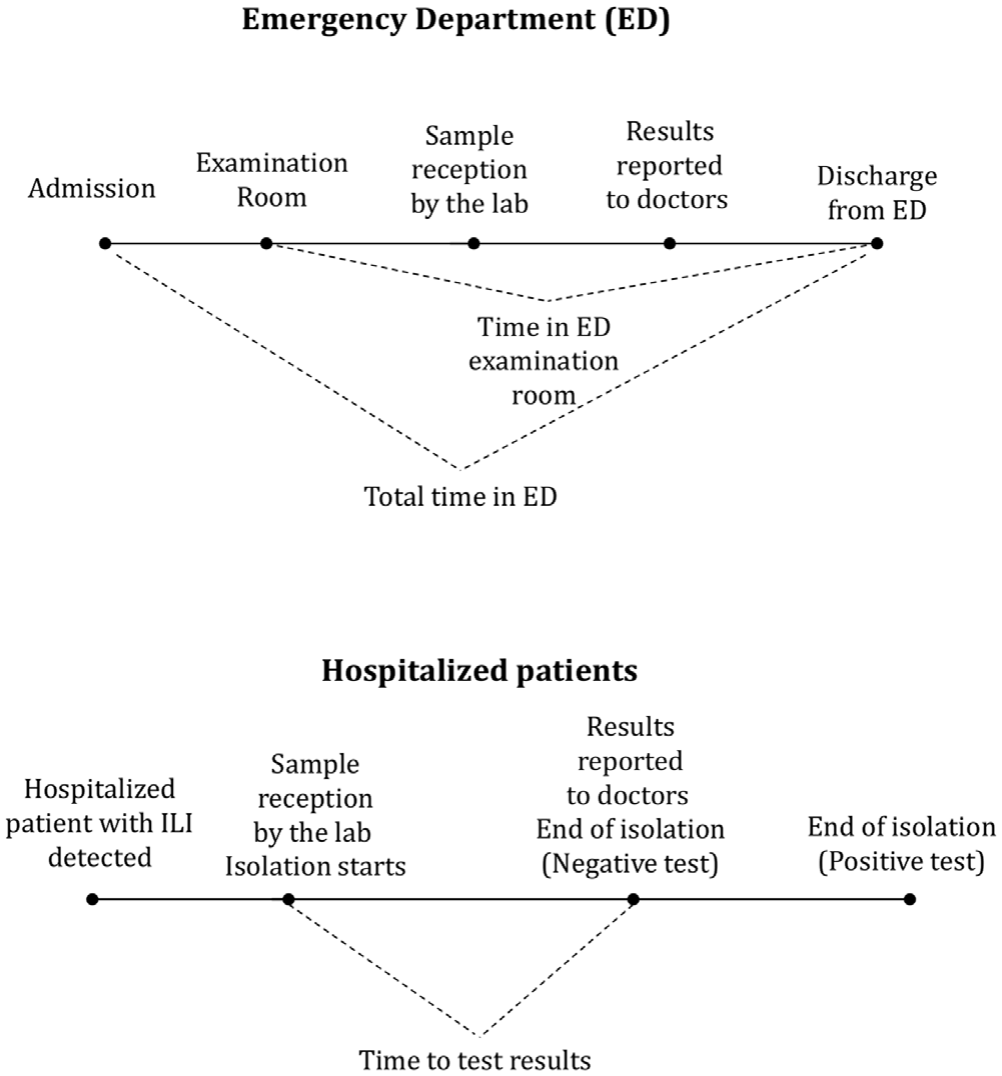
Time measures used for the cost analysis.

Descriptive statistics were used to summarize the characteristics of both ED and hospitalized patients. We collected data on demographic and clinical characteristics of patients, influenza test results and time to delivery of test results. Additionally, for ED patients we collected data on triage category (triage I and V are the most and least urgent ED patients, respectively) and time spent in the ED examination room (Fig 1). Time spent in the examination room by all ED patients (ILI and non-ILI) in both study periods was also measured to analyze if variables not related to influenza testing affected times in the ED department.

As to hospitalized patients, we attempted to measure the isolation time but the hospital does not have records of the time that patients spent in isolation. As a surrogate measure, it was assumed that patients began the isolation period at the same time the samples were received by the hospital laboratory. This assumption was based on the fact that in our hospital isolation of influenza suspect patients and collection of samples are performed simultaneously. The end of the isolation period was assumed to occur when a negative test result was reported or five days after the beginning of isolation in the case of a positive test.

The characteristics of the patients tested in 2013 and 2014 were compared using the Wilcoxon rank-sum test and the Fisher’s exact two-sided test for continuous and categorical variables, respectively. A probability threshold of 5% was defined to identify statistically significant differences.

The mean costs incurred by ED and hospitalized patients were estimated separately and were evaluated from the perspective of the Hospital Clinic of Barcelona. Costs not directly related to influenza testing and treatment were not considered.

The costs for ED patients considered the resources used for ILI patients during their stay in the ED examination room, including the price of reagents, technical staff in charge of test processing, use of antivirals and the cost of the use of the ED examination room, including disposables (masks, gloves, and clothing) and the cost of health care personnel (doctors and nurses). This latter cost was calculated according to the mean time spent by a patient in the ED examination room. To do so, a proportional-hazard (Weibull) regression model was implemented to estimate the time spent in the ED examination room adjusted by the patients’ characteristics [17]. The covariates included in the regression were age, gender, number of risk factors, triage category, test result, and type of test (in-house PCR or Xpert^®^ Flu). Due to the low number of observations of some of the triage categories, triage I and II were classified into a single category as were categories IV and V. Estimates from the regression analysis of time spent in the ED examination room were used for the calculation of the mean cost per ED patient tested for influenza.

All ED patients included in the study received antiviral treatment because one of the conditions for inclusion was to be at high risk of influenza-related complications [12]. Treatment with oseltamivir was assumed to start at the time of the reception of samples for the diagnosis of influenza by the hospital laboratory and stopped upon the reporting of a negative test result. In the case of a positive test, treatment was assumed to be of five days, which is considered to be the standard duration of treatment with oseltamivir [18]. Since patients in the ED are not isolated, no additional isolation costs were included.

The costs associated with hospitalized patients considered the use of resources when a case of ILI was detected and placed in isolation. These resources included reagents, technical staff, antivirals, disposables used by health care personnel and those due to family visits. Hospitalization costs also took into account the extra nursing time for isolated patients compared with non-isolated hospitalized patients. Isolated patients do not require extra physician dedication and therefore no supplementary cost in this respect was added. Treatment with oseltamivir was assumed to match the isolation time.

Unit costs and other parameter values used for the calculation of the mean baseline cost and for the sensitivity analysis were obtained from hospital records and are presented in Table A in S1 File. These costs include the cost of an ED stay per day, the cost of disposables used in the isolation room, laboratory technician cost per hour, and technician time needed to process each test, antiviral treatment, and reagents. The price of Xpert^®^ Flu was obtained from Cepheid.

A deterministic sensitivity analysis was performed to assess the impact of varying resource use and other relevant parameters while the other parameters remained constant at their baseline values. Variables with the highest impact on costs were identified. The results are presented as a Tornado diagram [19]. Statistical analyses were performed using STATA 13.1 (StataCorp, College Station, Texas USA).

## Results

### Test results

A total of 1057 patients were included in the study. Of the 366 samples from 2013 that were processed only by in-house real-time PCR, 97 (26.5%) were positive. The 691 samples from 2014 were processed by both in-house real-time PCR and the Xpert^®^ Flu assay; 185 (27%) specimens were positive by both methods, 500 (72%) specimens were negative and 6 (0.9%) were discordant. The Kappa agreement coefficient was 97.8% (p<0.0001). See Tests Results A in S1 File.

### Patient characteristics and waiting times

Table 1 shows the characteristics of the ED (n = 550) and hospitalized patients (n = 507). No statistically significant differences were observed in age, mean number of risk factors or the influenza prevalence rate between 2013 and 2014. In the ED, a significantly greater number of males were tested in 2014 (p = 0.016). In that year, the distribution of patients by triage category was more concentrated in those requiring urgent treatment compared to 2013 (p = 0.009).

**Table 1:** Characteristics of Emergency Department and hospitalized patients. ED: Emergency Department. SD: Standard Deviation.

	2013 (N = 366)	2014 (N = 691)
**ED Patients**	n = 130	n = 420
Age, mean years (SD)	59.1 (18.8)	61.8 (17.9)
Male*, n (%)	59 (45.4%)	242 (57.6%)
Number of risk factors, mean (SD)	2.08 (1.38)	1.94 (1.27)
Influenza-Positive, n (%)	54 (41.5%)	139 (33.1%)
Triage level*, *n (%)*		
I	0 (0%)	8 (1.9%)
II	23 (17.7%)	87 (20.7%)
III	90 (69.2%)	305 (72.6%)
IV	14 (10.8%)	14 (3.3%)
V	3 (2.3%)	6 (1.4%)
**Hospitalized Patients**	n = 236	n = 271
Age, mean years (SD)	62.4 (21.1)	62.4 (19.0)
Male, n (%)	146 (61.9%)	146 (53.9%)
Number of risk factors, mean (SD)	2.71 (1.12)	2.58 (1.26)
Influenza-Positive, n (%)	43 (18.2%)	52 (19.2%)

* Statistically significant differences between 2013 and 2014 (α = 5%)

The time spent in the ED examination room in 2014 was shorter than in 2013 [20.7 hrs. vs. 28.1 hrs. (p = 0.003)] as was the time to influenza test results [4.3 hrs. vs. 27.1 hrs. respectively (p<0.001)] (Table 2). As to hospitalized patients, the mean time for influenza test results was 5.8 hours in 2014 vs. 29.5 hours in 2013 (p<0.001).

**Table 2:** Time in the ED examination room and time to influenza test results, mean hours (SD). ED: Emergency Department. SD: Standard Deviation.

	2013	2014	Difference
Time in ED examination room	28.1 (25.0)	20.7 (20.2)	-7.4*
Time to test results (ED patients)	27.1 (18.5)	4.3 (3.2)	-22.8*
Time to test results (hospitalized patients)	29.5 (20.3)	5.8 (8.1)	-23.7*

* Statistically significant differences between 2013 and 2014 (α = 5%)

The mean time spent in the ED examination room spent by all patients (ILI and non-ILI) was 10.3 hours in January-March 2014 versus 11.4 hours in January-March 2013 (p = 0.85). Therefore, no statistically significant differences were observed in the waiting times between the two periods for the whole group of ED patients.

Table 3 shows the results of the regression analysis for the time spent by ILI patients in the ED examination room showing statistically significant covariates. The number of risk factors, the urgency of the patients, and a negative test result were associated with longer stays. The use of Xpert^®^ Flu was associated with a shorter waiting time in the ED. The adjusted time reduction of the use of the Xpert^®^ Flu versus the in-house PCR test was estimated to be 9.1 hours (95% CI: -14.7; -3.5).

**Table 3:** Regression analysis of the time spent in the ED examination room. Weibull regression. ED: Emergency Department

	**Hazard Ratio**	**p value**	**95% Confidence Interval**	
Number of risk factors	0.923	0.039	0.856	0.996
Triage I or II	0.505	0.011	0.297	0.856
Triage III	0.589	0.037	0.358	0.967
Triage IV or V	1.000	—	—	—
Flu-Positive	1.345	0.002	1.117	1.620
Xpert^®^ Flu	1.487	<0.001	1.198	1.845
	**Mean Marginal Effect**	**p value**	**95% Confidence Interval**	
Xpert^®^ Flu (hours)	-9.1	0.002	-14.7	-3.5

In the base case, the mean costs per ED patient were 294.6€ for Xpert^®^ Flu and 397.7€ for the in-house PCR test (Table 4). Savings associated with Xpert^®^ Flu (103.1€) were almost fully explained by the shorter time spent in the ED examination room. The mean costs for hospitalized patients were 143.7€ and 207.9€ for the Xpert^®^ Flu and the in-house PCR test, respectively. Savings in unnecessary isolation of negative patients (disposables and nursing) explain the difference.

**Table 4:** Mean cost per ED and hospitalized patient. ED: Emergency Department. (a) Assuming a 5-day period of antiviral treatment and isolation. (b) Assuming length of antiviral treatment equal to time to test results. (c) Assuming length of isolation time equal to time to test results. (d) Assuming 1 nurse every 6 isolated patients (versus 9 in non-isolated patients).

**ED Patients**			
	**Xpert^®^ Flu (€)**	**In-house PCR (€)**	**Difference (€)**
**Test**	**46.1**	**41**	**5.1**
Reagents	45	20	25.0
Laboratory technical staff time	1.1	21	-19.9
**Antiviral treatment**	**12.5**	**16.7**	**-4.2**
Positive	31.5	31.5	0.0
Negative	3.2	9.5	-6.3
**ED examination room**	**236**	**340**	**-104.0**
**Total per ED patient**	**294.6**	**397.7**	**-103.1**
**Hospitalized patients**			
	**Xpert^®^ Flu (€)**	**In-house PCR (€)**	**Difference (€)**
**Test**	**46.1**	**41**	**5.1**
Reagents	45	20	25.0
Laboratory technical staff time	1.1	21	-19.9
**Antiviral treatment (mean)**	**8.5**	**13.6**	**-5.1**
Positive (a)	31.5	31.5	0.0
Negative (b)	3.2	9.5	-6.3
**Disposables (mean)**	**46.1**	**79.3**	**-33.2**
Positive (a)	200	200	0.0
Negative (c)	10	51	-41.0
**Extra nurse dedication (d)**	**42.9**	**74.0**	**-31.0**
Positive (a)	186.3	186.3	0.0
Negative (c)	9.3	47.6	-38.3
**Total per hospitalized patient**	**143.7**	**207.9**	**-64.2**
Positive	463.9	458.8	5.1
Negative	68.6	149.1	-80.5

**Fig 2:**
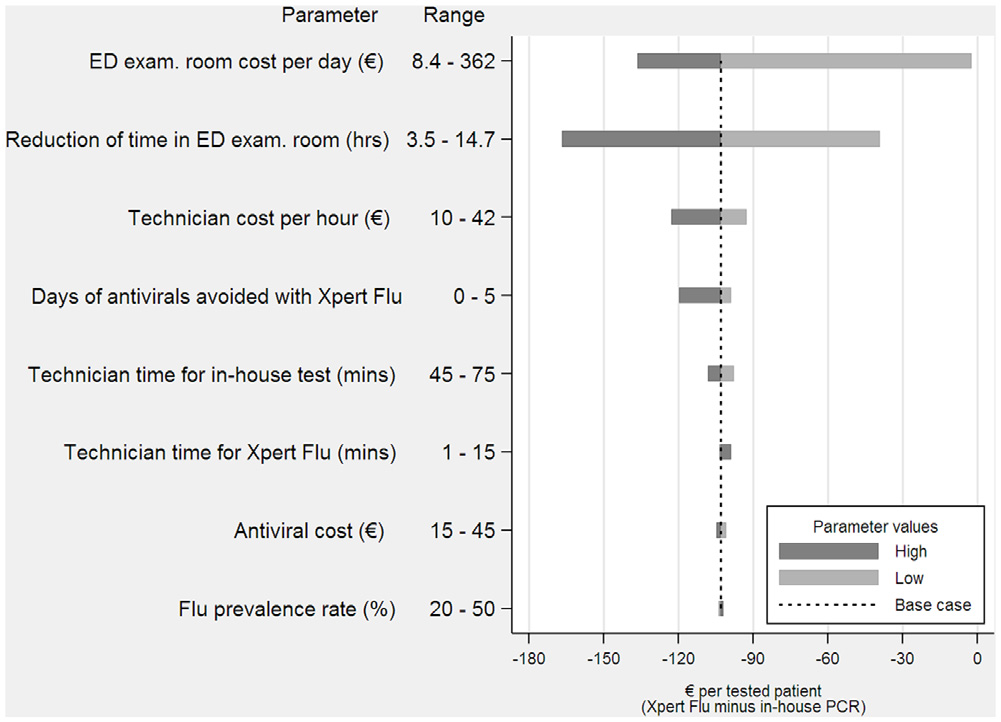
Incremental cost for Emergency Department patients (Xpert^®^ Flu minus in-house PCR).

**Fig 3:**
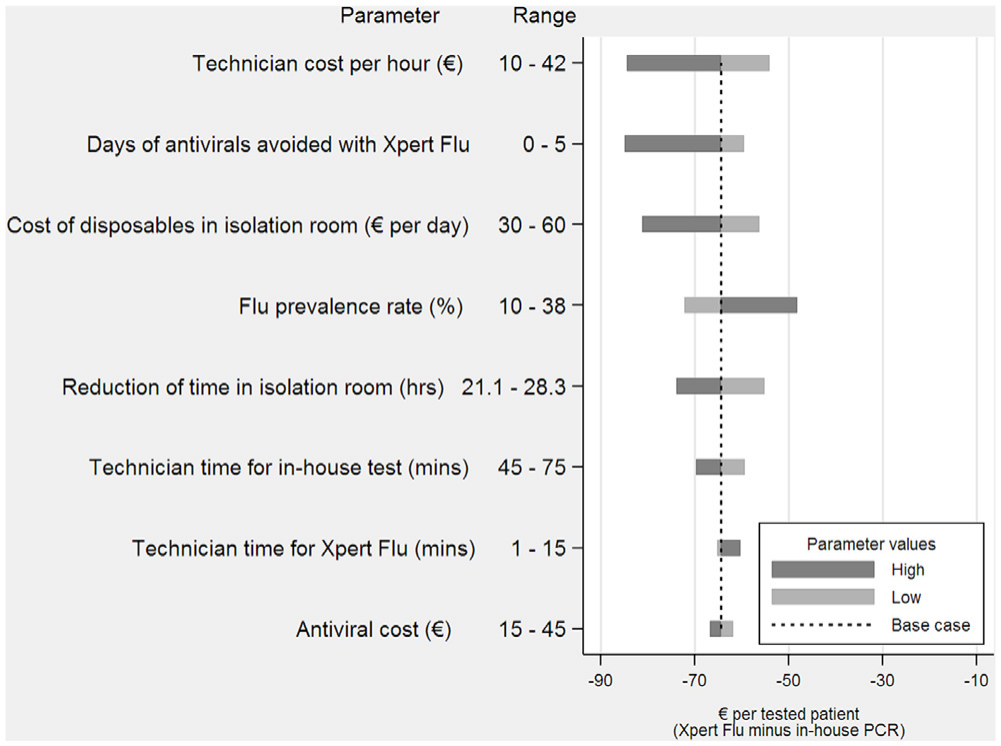
Incremental cost for hospitalized patients (Xpert^®^ Flu minus in-house PCR).

The sensitivity analysis showed that the results were robust with no single variable modifying the finding that Xpert^®^ Flu was less costly compared to the in-house PCR test (Figs 2 and 3). In the case of ED patients, the most sensitive results were found in the variations in the cost per day of the ED examination room. The lowest value assumed for this parameter was 8.3€ per day, which only accounted for the daily cost of disposables used in the ED (it did not consider the costs of health care staff). This scenario is relevant in cases in which health care staff has a fixed-cost. In this case, Xpert^®^ Flu continued to be less expensive, but the savings per ED patient decreased to only 2.4€. The costs were also sensitive to the estimated reduction in the time spent in the ED examination room. However, even assuming the lowest reduction in waiting time, the savings per patient tested were still considerable (39€ per ED patient). The sensitivity analysis did not affect the main results for hospitalized patients either. Savings were lowest when the influenza prevalence rate among these patients was high (38% versus 18.2% in the base case scenario) in which case savings per patient were still important (48€ versus 64.2€ in the base case).

## Discussion

To our knowledge this is the first economic analysis evaluating the impact of rapid influenza testing on waiting times and costs for patients with a high risk of complications in the ED and on isolation costs for hospitalized patients with ILI.

In this study Xpert^®^ Flu showed a high sensitivity and specificity, which was similar to previous studies [20], and its use reduced the time required to detect influenza virus compared to our in-house PCR test. Although the reagents of Xpert^®^ Flu were more expensive than those of our in-house PCR, the new technique increased laboratory efficiency by cutting hands-on time and operational steps. Therefore this technique significantly reduced the time between the reception of samples by the laboratory and the reporting of test results to doctors. As a consequence, waiting times in the ED and isolation of hospitalized patients with ILI as well as associated costs were reduced.

Patients tested with Xpert^®^ Flu spent a mean of 20.7 hours in the ED examination room, which was 7.4 hours less than that spent by patients tested with our in-house PCR assay. Nevertheless, according to the differences observed in triage categories, patients with ILI attending the ED in 2014 had a more serious health condition than those of 2013. On adjustment for patient characteristics, the difference in waiting time between the two groups of patients was 9.1 hours. The results also showed that a reduction in waiting time was not observed in non-ILI patients over the study period. This finding is important since the occurrence of a major event in the ED affecting the speed of discharge in 2014 would have affected all the patients visiting the ED. However, the data suggest that no such event took place.

Xpert^®^ Flu also introduced benefits in efficiency in the management of hospitalized patients because early negative results reduced unnecessary isolation. Assuming that the time to test results was equal to the time that hospitalized influenza-negative patients with ILI spent in isolation, a reduction of about 24 hours in isolation time was observed in these patients.

We estimate that Xpert^®^ Flu reduced costs of testing and treating patients with ILI by 103€ (or $113) per ED patient and 64€ ($70) per hospitalized patient. For ED patients, the cost decrease was mainly due to savings associated with a reduced need for health care staff per patient. For hospitalized patients, the cost reduction was equally explained by savings in nurse dedication for isolated patients and by a reduction in the use of disposables needed to enter the isolation room. Additionally, early diagnosis helped to decrease the use of antivirals in patients that did not need these drugs.

The results were robust to a number of parameter changes. The variable that introduced the highest uncertainty in the estimates was the cost associated with ED stay. In the base case analysis this cost accounted for disposables and the cost per hour of health care staff. In a context in which the health care staff is considered to be a fixed-cost (i.e., a cost not affected by an ED room being occupied by a patient or not) savings would have been substantially lower. Variations in the remaining parameters used for estimation did not affect the main results. Similarly, no single parameter changed the main findings regarding savings for hospitalized patients with ILI.

Xpert^®^ Flu has other advantages that cannot be measured in monetary terms but which are also important for patient care, such as the quality of service to patients thanks to the reduced waiting time in the ED. Moreover, physicians informally declared their satisfaction of having a test that quickly provided an accurate diagnosis. Besides, rapid diagnosis during the influenza season can reduce the exposure of other patients and staff in the ED to the influenza virus. Based on these considerations, our hospital implemented the use of Xpert^®^ Flu during the 2015 influenza season for the diagnosis of patients with ILI attending the ED and at risk of influenza complications and for hospitalized patients with ILI.

This study has several limitations. First, it compared data obtained in two different years, thereby raising the possibility of sample bias. In particular, a hypothetical change of management or in the organizational structure in the ED between the two years of analysis could have caused a reduction in the time of discharge of ED patients. Nevertheless, no significant change in ED time reduction for the whole group of ED patients (ILI and non-ILI) was observed between the two periods. Another potential drawback is that records of isolation time of hospitalized patients were not available and thus, the study had to rely on indirect measures of isolation time. This is a potential source of bias (positive or negative) in the estimated cost reduction. Similarly, the use of antivirals was also based on indirect measures. However, the sensitivity analysis showed that the results were robust to changes in the assumption about a reduction of isolation time and the days of use of antivirals avoided. Finally, the study was based on prices and costs incurred by our hospital which may differ from those in other contexts or countries. However, the main results of our research were robust to a number of plausible variations in prices and parameters. Therefore, despite these limitations, the present study provides valuable information for different settings.

## Conclusions

In summary Xpert^®^ Flu significantly reduced the time to discharge of patients attending the ED and also decreased the isolation time of hospitalized patients. As a consequence, the technique had a significant impact on costs due to optimization of patient management.

## Supporting Information

S1 File (DOCX)

S1 Dataset ED patients. (DTA)

S2 Dataset Hospitalized patients. (DTA)
