# Supplementary material for: Economic Impact of a New Rapid PCR Assay for Detecting Influenza Virus in an Emergency Department and Hospitalized Patients
Source: PLoS One. 2016 Jan 20;11(1):e0146620. doi: 10.1371/journal.pone.0146620 (PMC4720278; doi:10.1371/journal.pone.0146620)
Supplement: S1 File — (DOCX) [file pone.0146620.s001.docx]

# Economic impact of a new rapid PCR assay for detecting influenza virus in an Emergency Department and hospitalized patients

Marcelo Soto^1^, Laura Sampietro-Colom^2^, Anna Vilella^3,4^, Efraín Pantoja^3^, María Asenjo^5^, Ruth Arjona^4,6^, Juan Carlos Hurtado^4,6^, Antoni Trilla^3,4^, Míriam José Alvarez-Martínez^4,6^, Aurea Mira^7^, Jordi Vila^4,6^, María Angeles Marcos^4,6^

^1^Fundació Clínic per a la Recerca Biomèdica, Barcelona, Spain; ^2^Health Technology Assessment Unit, Hospital Clínic, University of Barcelona, Barcelona, Spain; ^3^Public Health Department, Hospital Clínic, University of Barcelona, Barcelona, Spain; ^4^ISGlobal Barcelona Institute for Global Health, Barcelona, Spain; ^5^Emergency Department, Hospital Clínic, University of Barcelona, Barcelona, Spain; ^6^Department of Clinical Microbiology, Biomedical Diagnostic Centre (CDB), Hospital Clínic, University of Barcelona, Barcelona, Spain. ^7^Biomedical Diagnostic Centre (CDB), Hospital Clínic, University of Barcelona, Barcelona, Spain.

**S1 File**

| **Supporting information Table A.** Unit costs and parameter values | | | |
| --- | --- | --- | --- |
|  | **Base case** | **Low Value** | **High Value** |
| ***Unit costs*** |  |  |  |
| ED examination room cost (€/day) (a) | 273.6 | 8.4 | 362 |
| Technician cost (€/hour) | 21 | 10 | 42 |
| Antiviral Cost (€/5 days) | 31.5 | 15 | 45 |
| Disposables isolation room (€/day) | 40 | 30 | 60 |
| Xpert^®^ Flu reagents (€/test) | 45 | -- | -- |
| In-house PCR reagents (€/test) | 20 | -- | -- |
|  |  |  |  |
| ***Other parameters*** |  |  |  |
| Reduction in time spent in ED examination room (hrs) | 9.1 | 3.5 | 14.7 |
| Reduction in isolation time for hospitalized patients with negative test (hrs) | 24.7 | 21.1 | 28.3 |
| Technician time, Xpert^®^ Flu (min) | 3 | 1 | 15 |
| Technician time, in-house test (min) | 60 | 45 | 75 |
| Percentage of positive tests (ED patients) | 33% | 20% | 50% |
| Percentage of positive tests (hospitalized patients) | 19% | 10% | 38% |
| Days of antivirals avoided by patients with negative tests | 1 | 0 | 5 |
| (a) Staff + disposables ED: Emergency Department. |  |  |  |

**Molecular detection A**

The nasopharyngeal and oropharyngeal swabs collected from the patients were placed in the same tube containing 3 ml of viral transport medium (VTM) and immediately sent to the Microbiology Laboratory for virological analysis.

In 2014, all the samples obtained were prospectively analyzed in parallel by in-house PCR and the Xpert^®^ Flu assay. Samples analyzed by the Xpert^®^ Flu assay (Cepheid, Sunnyvale, CA), were processed upon arrival to the Microbiology laboratory 24-hours a day 7-days a week. Three hundred microliters of the sample were added to the Xpert cartridge according to the manufacturer´s recommendations and the assay was performed in the Cepheid GeneXpert Instrument System.

In-house PCR was performed daily on working days (Monday–Friday) from 9 am to 3 pm, and the samples were kept at 4ºC. Three hundred microlitres of each specimen were taken for total nucleic acid extraction and eluted in 25 μl of RNase-free elution buffer using the automatic QIAsymphony system (Qiagen, Hilden, Germany) according to the manufacturer´s instructions. Subsequently, two specific one-step in-house multiplex real-time PCR were used for typing A/B influenza virus and subtyping (H1/H3/H5/pdmH1N1) influenza A virus as previously described using Stratagene Mx3000P (Stratagene, La Jolla, CA). The detection limits of the typing assay were 355 and 412 copies/ml of sample for influenza A and B viruses, respectively. The detection limits of the subtyping assay were 357, 430, 345 and 412 copies/ml of sample for seasonal H1, pandemic H1, seasonal H3, and avian H5, respectively. Specificity analysis was ensured testing RNA extracted from samples of the 2009 WHO External Quality Assessment Programme for the Detection of Influenza Virus by PCR. Discrepant results obtained by both methods in the prospective samples were confirmed by DNA sequence analysis.

For this study, the results of the nasopharyngeal and oropharyngeal samples obtained from ED and hospitalized patients with ILI from January 1 to March 30, 2013 were used as historical controls.

**Test Results A**

Of the 366 samples from 2013, 97 (26.5%) were positive by in-house PCR, 25 (26%) were influenza A virus (5 H3N2; 16 2009 H1N1 and 4 not subtyped) and 72 (74%) were influenza B virus.

Of the 691 samples from 2014, 185 (27%) were positive by both methods, (184 were influenza A virus and 1 was influenza B virus); 500 (72%) specimens were negative, and 6 (0.86%) were discordant.

The mean PCR threshold cycle (Ct) of the 185 positive samples was 21. Positive samples were further subtyped by the in-house real-time PCR assay as H3N2 (n=111, 60%), as 2009 H1N1 (n=46, 25%), and 27 (15%) were not subtyped. The 46 samples with influenza A 2009 H1N1 detected by the in-house PCR were also subtyped by the Xpert^®^ Flu assay. Of the 6 discordant samples, 5 were positive only by in-house PCR (3 H3N2, 2 non-typed), and 1 was positive for influenza B virus only by the Xpert^®^ Flu assay. This latter sample demonstrated a Ct value of 26 in the PCR assay and was confirmed by RNA sequence analysis. However, the mean Ct value of the 5 Xpert flu negative samples was > 34, and these were not confirmed by RNA sequence analysis suggesting that low viral load might be the reason for discrepancy. The overall sensitivity and specificity of Xpert^®^ Flu were 97.4% (95% CI: 94.0 –99.1) and 100 % (on-sided 97.5% CI: 99.2 – 100), respectively.

| **Supporting information Table B.** Comparative evaluation of the Xpert^®^ Flu assay and the in-house PCR test. | | | |
| --- | --- | --- | --- |
|  | Xpert^®^ Flu + | Xpert^®^ Flu - | Total |
| In-house PCR + | 185 | 5 | **190** |
| In-house PCR - | 1 | 500 | **501** |
| **Total** | **186** | **505** | **691** |
